# EU-Africa partnerships in health research from 2014 to 2023: Outputs and lessons learnt

**DOI:** 10.1016/j.ijid.2025.108331

**Published:** 2026-03

**Authors:** Debora Bade, Andreia Coelho, Dominika Jajkowicz, Henrique Pinheiro, Thomas Nyirenda, Michael Makanga, Pauline Beattie

**Affiliations:** 1 European & Developing Countries Clinical Trials Partnership (EDCTP), The Hague, The Netherlands; 2 Analytical Services, Elsevier, Montreal, Canada; 3 EDCTP Association, Cape Town, South Africa; 4 Global Health EDCTP3 Joint Undertaking, Brussels, Belgium; 5 United Nations University World Institute for Development Economics Research, Helsinki, Finland

**Keywords:** Development of medical interventions for the main infectious diseases affecting sub Saharan Africa, Research financing, Research equity, Clinical trials, North-South and South- South partnerships, Clinical research capacity

## Abstract

**Objectives:**

The European & Developing Countries Clinical Trials Partnership (EDCTP) supports clinical research partnerships between countries in Europe and sub-Saharan Africa. A bibliometric analysis of outputs from the second EDCTP program, EDCTP2, was undertaken to assess its contribution to international collaboration, advancement of scientific knowledge and policy uptake.

**Methods:**

1429 papers acknowledging EDCTP2 support published between 2014 and the end of 2023 were compared with all publications within the scope of EDCTP2 and with subsets acknowledging other funders.

**Results:**

86.3% of EDCTP2 publications included at least one author from sub-Saharan Africa (SSA) and 62% included authors from both European and SSA countries. SSA researchers were first or last authors on 71% of EDCTP2 papers and women researchers from SSA were lead authors on 33% of EDCTP2 papers. Collaborations across several European–African and African–African country pairs were over-represented in EDCTP2 outputs. EDCTP2 papers were more likely than those from other funders to focus on the affordability and accessibility of medical interventions and on populations with unmet medical needs typically excluded from clinical studies, such as children, pregnant women and people with co-infections and co-morbidities. We corroborated that EDCTP publications were influencing policy.

**Conclusion:**

Although the findings reflect outputs part-way through the EDCTP2 program, they provide encouraging signs that the EDCTP2 program is meeting its objectives on promoting international collaboration, widening access to interventions, and delivering public health benefits to populations in sub-Saharan Africa.

**Funding:**

This work was supported by the UK National Institute for Health and Care Research (NIHR) and the EDCTP2 program, supported by the European Union.

## Highlights

• EDCTP2 funding led to North- South partnerships extending beyond historical links.
• Authors from sub-Saharan Africa led more than two third of EDCTP2 publications.
• Women account for 40% of co-authors and led one third of EDCTP2 outputs.
• Vulnerable populations are strongly represented in EDCTP2 papers.
• EDCTP2 outputs are influencing global and national policy.

## Introduction

The second EDCTP program, EDCTP2, was launched in 2014 as part of the European Union’s Horizon 2020 funding program. Calls for proposals were launched from 2014 to 2020, and grants were awarded between 2016 and 2022. Its key objectives were to support international clinical research collaborations to advance the development of medical interventions for the main infectious diseases affecting SSA and to strengthen local clinical research capacity. A successor program, the Global Health EDCTP3 Joint Undertaking, was launched in 2021.

The EDCTP2 program supported clinical trials and non-interventional studies to advance the development and implementation of drugs, vaccines, prognostics and diagnostics for diseases such as HIV/AIDS, tuberculosis (TB), malaria, neglected infectious diseases (NIDs), diarrheal diseases, lower respiratory tract infections (LRTI), and emerging and re-emerging infections of particular relevance to SSA. EDCTP2 also provided funding to strengthen the ethical, legal and regulatory environment for clinical research in SSA countries [1].

The EDCTP2 program operated according to a set of core principles, including a commitment to equitable partnerships, at all levels, from program governance to the management of individual grants [2]. Also, EDCTP2 has a particular focus on late-stage clinical trials, product-focused implementation research, and populations often excluded from pivotal trials, such as pregnant and lactating individuals, children, and people with co-infections or comorbidities.

The EDCTP2 secretariat commissioned a bibliometric analysis of publications acknowledging EDCTP2 funding, to explore:

• Patterns of collaboration between researchers in Europe, SSA and elsewhere.
• Authorship trends and representation of researchers from SSA and women researchers.
• The focus on two key themes, involvement of excluded groups in clinical studies and affordability/accessibility.
• The advancement of scientific knowledge.
• Evidence of policy uptake.

Scientific publications are the primary route to disseminate evidence generated in research projects.

Bibliometric analysis is a powerful way to explore the outputs and contributions of funded research grants, but has significant limitations when used as a sole measure of research impact [3]. Wherever possible, we used a comparative approach.

## Methods

Data curation and analysis were carried out by Science-Metrix. The objectives of the study were defined by the EDCTP2 secretariat.

### Identification of EDCTP2 publication set

Publications resulting from EDCTP2 grant funding are collected routinely. The resulting list of publications was matched to the Scopus database via digital object identifiers (DOIs) and titles. Additional publications were identified in Scopus via EDCTP2 grant-specific identifiers listed in acknowledgement sections. The bibliometrics analysis included publications from 1 January 2016 to 31 December 2023. This publication dataset is referred to as “EDCTP2 publications” hereafter.

### Comparator outputs

The EDCTP2 publication set was compared with a range of other publication sets filtered to include only papers in the disease areas of EDCTP2 interest:

• Separate datasets for each of the most common EDCTP2 co-funders.
• Separate datasets for specific geographic locations.

### Collaboration

Country involvement was assessed by reference to the affiliations of all co-authors. Fractional counting that weights all publications equally by fixing the total number of collaborations at one for each publication was used to minimize the impact of single highly-collaborative publications. Comparisons with non-EDCTP2-funded outputs were used to identify individual countries and pairs of countries over-represented in EDCTP2 outputs.

### Authorship

First and last authors were classified as lead authors. The gender of authors was inferred based on the NamSor database (https://namsor.app).

### Content classification

Papers were categorized into the disease areas of interest to EDCTP2 based on the analysis of sets of keywords derived from the Emtree thesaurus (https://www.elsevier.com/en-gb/products/embase/emtree) and their presence in titles, abstracts or curated terms. A large language model (LLM) was trained to identify additional papers with similar content but without the keywords in titles, abstracts or curated terms [4].

Frequently excluded populations: Populations frequently excluded from clinical trials include women, especially pregnant/lactating individuals, newborns, children and adolescents; and people with co-infections and comorbidities. These terms were used as keywords to filter outputs. Keywords were checked in context to identify false positives. Such keywords were either excluded from the filter or combined with other keywords to increase the precision of the set of publications identified.

Affordability and accessibility: The initial keywords used were cost, supply, affordability, accessibility, equitability and acceptance. Then a LLM was used to ensure that filtered publications related to a medical intervention (broadly defined, such as tools for diagnosis, preventive medicine, medical treatments and devices, and tools or methods for epidemic outbreak detection).

We first calculated the (weighted) average shares (%) across each research area, year, and type of document and then normalized the actual share by dividing it by the expected share.

### Scientific knowledge

Scholarly citations of EDCTP2 publications published between 2016 and 2021 up to the end of 2023 were compared with those of the comparator datasets described above. Two measures were used to assess citation rates: the average of relative citations (ARC), a score normalized by the average citation count of all papers published in the same year, subfield and document type [5]; and the citation distribution index (CDI). The CDI includes the sum of the weighted share of each decile of a distribution of publications, ranked by citation count [6]. Scores higher than zero indicating above-average performance.

### Policy uptake

This analysis examined how often publications were cited in policy-relevant literature indexed in Overton, the largest database of policy-related documents [7].

## Results

### EDCTP2-supported papers dataset

We identified 1429 EDCTP2 outputs published between 2016 and 2023. These were assigned to thematic areas used by EDCTP2 for administrative purposes and compared with the funding allocations in these areas (Table 1). Notably, the ‘emerging diseases’ category accounted for a relatively high proportion of outputs, 19.7% of the total, despite receiving 10.7% of EDCTP2 funding. This may reflect the need for rapid research and publication.

**Table 1:** Overview of EDCTP2 grant funding by disease areas and the share of publications linked to these grants. Grants may focus on more than one disease area but are assigned to a single primary area for administrative purposes.

EDCTP2 grant classification	direct grant funding (€m)/(% share)	No. of publications (% share)	% share (allocated only)	% of open access
Diarrheal diseases	54.1 (6.5)	18 (1.3)	1.4	100
Emerging diseases	88.8 (10.7)	282 (19.7)	21.7	96
HIV and associated infections	132.1 (15.9)	220 (15.4)	16.9	95
Lower respiratory tract infections	42.6 (5.1)	21 (1.5)	1.6	95
Malaria	157.2 (18.9)	130 (9.1)	10.0	95
Neglected infectious diseases	80.0 (9.7)	117 (8.2)	9.0	95
Tuberculosis	215.5 (26.0)	345 (24.1)	26.5	94
Capacity building and training	11.2 (1.4)	32 (2.2)	2.5	100
Ethics and regulatory	15.9 (1.9)	14 (1.0)	1.1	100
EDCTP Regional Networks	30.0 (3.6)	114 (8.0)	8.8	95
Other/no primary disease	1.1 (0.1)	7 (0.5)	0.5	100
Grants not identified	NA	187 (13.1)	–	99
**TOTAL**	**828.5**	**1429 (100)**	100	**96**

13.1% of papers could not be assigned to a specific grant. There is a high correlation between the amount of funding spent on a specific disease area and the number of assigned publications (Pearson correlation coefficient=0.82). However, only 130 (9.1%) of publications were linked to Malaria focused grants despite a direct funding commitment of 157.2 **€**M (18.9%). For the latter, this likely reflects the award of large collaborative grants that had not yet generated significant outputs by the end of 2023. 96% of EDCTP2 publications were open access (Table 1), compared with the global average of 65% in EDCTP2’s areas of interest.

### Patterns of collaboration

**Figure 1:**
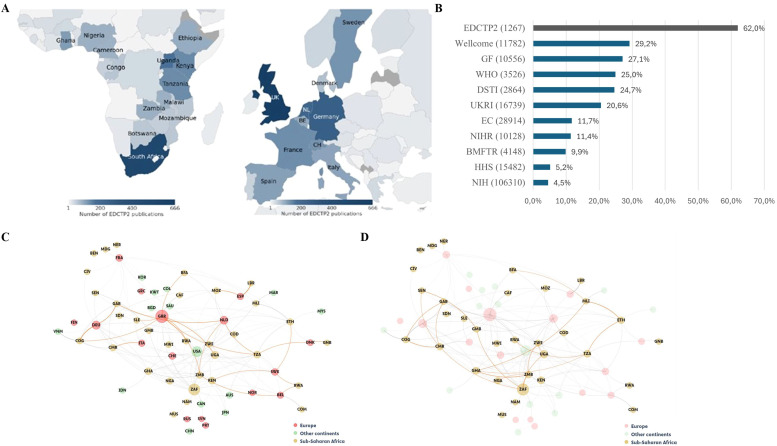
(a) Representation of authors from sub-Saharan African and European countries on EDCTP2 publications (2016-2023 outputs). Only countries represented on more than 50 EDCTP2 publications are shown. (b) Share of publications including authors from both Europe and SSA by funding agency. The total number of publications from 2016-2023 included in the founder specific dataset is indicated in brackets. GF: Gates Foundation; WHO: World Health organization; DSTI: Department of Science, Technology and Innovation (South Africa); UKRI: UK Research and Innovation; EC: European Commission; NIHR: National Institute for Health and Care Research (UK); BMFTR: Bundesministerium für Forschung, Technologie und Raumfahrt (Federal Ministry of Research, Technology and Space, Germany); HHS: Department of Health and Human Services (USA); NIH: National Institutes of Health (USA). (c,d) Graphical representation of the relative frequency of country pairs overrepresented in EDCTP2 publications. (c) Europe-SSA country collaborations overrepresented (≥50 times) in EDCTP2 publications are highlighted in orange. (d) Collaborations between sub-Saharan Africa countries with a relative frequency of ≥25 time are depicted in orange. See https://www.iso.org/obp/ui/#search for three-letter country codes.

The UK, Germany and the Netherlands were the three European countries most often represented in EDCTP2 publications (Figure 1a); 46% of publications included an author affiliated with an institution located in the UK. South Africa, Uganda and Kenya were the three SSA countries most frequently represented (Figure 1a); 36.3% of EDCTP2 publications involving an author affiliated with an organization in South Africa. A country’s representation on publications is in line with the amount of EDCTP2 funding received.

86.3% of EDCTP2 publications included at least one author from SSA, compared with 7.3% of papers in the global publications set; across funding agencies, the next highest proportion of publications including at least one author affiliated to an institution in SSA (excluding the Department of Science, Technology and Innovation (DSTI) in South Africa) was 41.4% for the Gates Foundation (GF). In addition, 33.6% of EDCTP2 publications included authors from two or more SSA countries, compared with 1.2% of global papers; across funding agencies, the next highest proportion was 15.0% for the World Health Organization (WHO).

62% of EDCTP2 publications included authors from both SSA and European countries. This was higher than the proportion seen in papers from other funders (Figure 1b).

To identify country pairings that are over-represented in EDCTP2 outputs, a comparison was made with all outputs with European or sub-Saharan African authors (Table 2, Figure 1 c/d), revealing where EDCTP2 funding has led to increasing collaborations between countries. Notable examples include the Republic of Congo and Gabon with Germany, Liberia with Spain, and multiple African country pairings, such as South Africa with Uganda and Zambia, Cameroon with South Africa and the Republic of Congo, and Ghana with Zambia.

**Table 2:** Selection of Africa–Europe and Africa–Africa country collaborations over-represented in EDCTP2 publications*.* The share (%) of publications corresponding to a country pair is indicated for EDCTP2 publications and publications in the remit of EDCTP2 involving authors from Europe or SSA.

Country A	Country B	No. of (fractional) collaborations	EDCTP2 (%)	EU or SSA (%)	EDCTP2/ (EU or SSA)
Congo, The Republic of	Germany	13.2	1.12%	0.001%	928.7
Gabon	Germany	10.3	0.88%	0.003%	278.1
South Africa	Uganda	9.3	0.79%	0.015%	52.2
South Africa	Zambia	7.2	0.61%	0.012%	51.2
Cameroon	South Africa	6.9	0.59%	0.018%	32.8
Ethiopia	Tanzania	6.5	0.56%	0.003%	161.1
Cameroon	Congo, The Republic of	4.4	0.37%	0.001%	284.2
Ghana	Zambia	3.9	0.33%	0.001%	349.2
Gabon	Netherlands	3.6	0.31%	0.001%	353.7
Liberia	Spain	3.5	0.30%	<0.001%	2120.2
Malawi	South Africa	2.6	0.22%	0.011%	20.0
Uganda	Tanzania	2.4	0.21%	0.006%	32.6
Cameroon	Gabon	2.3	0.20%	0.002%	93.9
Belgium	Zambia	2.0	0.17%	0.001%	206.0
Gambia	Senegal	2.0	0.17%	0.001%	283.7

### Authorship

**Figure 2:**
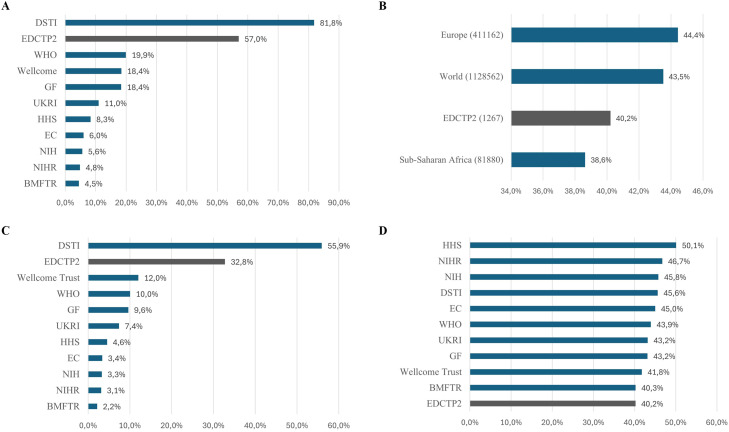
Characterization of co-authors contributing to EDCTP2 publications. (a) Percentage of co-authors affiliated to institutions located in sub-Saharan Africa on papers acknowledging different funders. (b) Percentage of women co-authors on EDCTP2 publications and those with authors from particular geographic regions. The total number of publications from 2016-2023 considered in each dataset is included in brackets (c) Percentage of women lead authors from sub-Saharan Africa on papers acknowledging different funders*.* (d) Percentage of women co-authors on papers acknowledging different funders.

On average, 57% of co-authors on EDCTP2 publications were affiliated with an institution in SSA (Figure 2a). This is higher than the equivalent for all other funders except DSTI.

In terms of lead authorship, 70.8% of EDCTP2 publications had a first or last author from a SSA country, compared with 5.4% of global publications. Excluding DSTI, the next highest percentage was 24.9% for the Wellcome Trust, followed by 24.2% for WHO and 21.6% for the Gates Foundation.

Women made up 40% of the authors of EDCTP2 papers. This is broadly the same as the proportion seen in all papers from SSA (Figure 2b) and slightly below the proportion seen in papers acknowledging other funders (Figure 2d).

Furthermore, women from institutions in SSA were lead authors on 33% of EDCTP2-supported papers (Figure 2c). This is higher than the equivalent figure for papers from all funders except the DSTI.

### Key themes: excluded populations and affordability/accessibility

**Figure 3:**
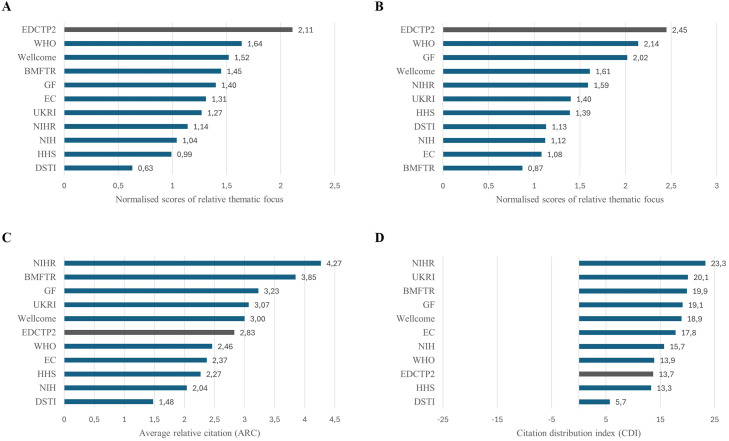
Relative focus on thematic areas of special interest to EDCTP2. (a) Relative focus on populations usually excluded from trials and (b) affordability and accessibility. Numbers represent normalized scores in comparison with all papers within the disease areas of EDCTP2 interest. (c/d) Citation scores of EDCTP2 publications compared to publications supported by other funders. (c) Average relative citation (ARC) score and (d) citation distribution index (CDI) for publications supported by EDCTP2 compared to other funders.

For excluded populations, the normalized score for EDCTP2 publications was 2.11, compared with 1.57 for SSA papers and 1.05 for European papers. This is also higher than the equivalent figure for papers acknowledging other funders (Figure 3a).

For affordability/accessibility, the normalized score for EDCTP2 publications was 2.45, compared with 1.84 for SSA papers and 1.01 for European papers. Again, this is higher than the equivalent figure for papers acknowledging other funders (Figure 3b).

### Scientific knowledge

The number of citations of a paper can provide a measure of its influence within the scientific community. The average relative citation (ARC) score for EDCTP2 papers was 2.83, indicating that they are almost three times more likely to be cited than other publications in the same field. This is a higher score than that seen for other well-known funders, including the US National Institutes of Health, WHO and European Commission, but below other key national and global philanthropic funders (Figure 3c).

When the citation distribution index (CDI) was applied, EDCTP2 papers still showed above-average performance, but their ranking relative to other funders fell slightly (Figure 3d), suggesting that a subset of EDCTP2 papers attracted a relatively high proportion of citations.

### Policy uptake

Referencing of publications in the Overton database was used as a proxy of policy impact. Because of the lag between publication and take up into policy outputs, this analysis focused on papers published between 2016 and 2021.

**Figure 4:**
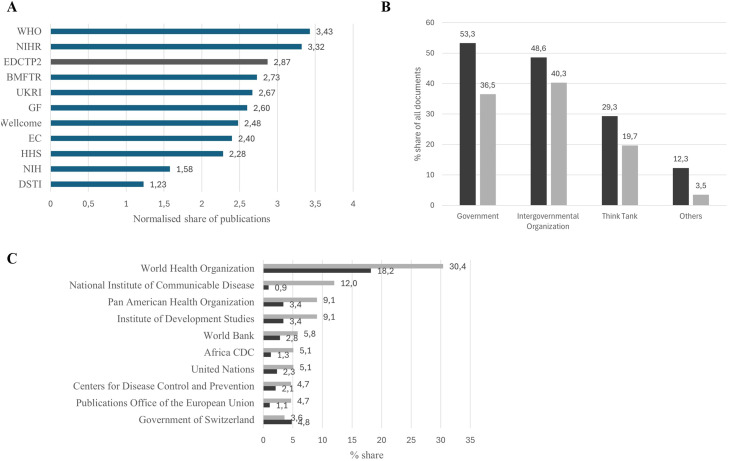
Uptake of EDCTP2 publication in the Overton database. (a) Likelihood of citation by publication in the Overton database of papers published in 2016-2021. (b/c) Share of EDCTP2 policy-cited publications cited by organization type (b) and citing organization (c). The share of EDCTP2 policy-cited publications is depicted in dark grey, whereas the share of citing documents is shown in light grey.

Overall, 24% of EDCTP2 publications were cited by policy-relevant documents. On average, an EDCTP2 publication was 2.87 times more cited than expected (Figure 4a). This placed EDCTP2 behind just two key funders, WHO and the UK NIHR.

An additional analysis examined the types of policy documentation citing EDCTP2-supported publications. This analysis focused on EDCTP2 outputs published between 2016 and the end of 2024 including a total of 1839 EDCTP2 publications. Of these, 276 EDCTP2 publications were cited by 970 policy-related documents.

134 (48.6% of the) cited papers supported by EDCTP2 were referenced by intergovernmental organizations such as WHO (Figure 4b/c). Such organizations published 391 (40.3% of the) documents referencing EDCTP2-supported work. WHO published 177 documents (share of 18.2%) citing EDCTP2 publications and referenced 84 (share of 30.4%) EDCTP2 supported publications cited in policy-documents.

National governments published 36.5% of referencing documents, with these outputs citing more than half (53.5%) of EDCTP2 cited papers.

Complementary routine monitoring of the EDCTP2 program by the EDCTP Association has identified multiple examples where data from EDCTP2 projects have already informed national and international policy and practice, including:

• *Arpraziquantel:* Data from this trial underpinned a positive scientific opinion published by the European Medicines Agency (EMA) in December 2023 for arpraziquantel to treat schistosomiasis in preschool-aged children [8]. This led to WHO prequalification in 2024 [9] and the first administration to young children in March 2025.
• *Fexinidazole:* Results from the HAT-r-ACC project led to a positive opinion from the EMA in December 2023 for fexinidazole as a treatment for human African trypanosomiasis (HAT) caused by *Trypanosoma brucei rhodesiense*, leading to a change in WHO treatment guidelines in October 2024 [10]
• *R21/Matrix-M:* WHO recommended the R21/Matrix-M malaria vaccine, drawing in part on data from the EDCTP2-funded phase II study [11].
• *High-dose liposomal amphotericin:* The AMBITION-cm trial [12,13] led to updated WHO guidance on treatment of HIV-associated cryptococcal meningitis and revision of national HIV treatment guidelines in the African countries that actively participated in the research [14].
• *Fixed-dose combination (FDC) of albendazole and ivermectin*: Data from this trial [15] led to a positive scientific opinion by the EMA in January 2025 [16] for an albendazole–ivermectin FDC to treat infections caused by soil-transmitted helminths and lymphatic filariasis in adults, adolescents and children aged 5 years or older.

## Discussion

This analysis of early outputs has demonstrated EDCTP2 has been highly effective at supporting international collaborations between and across countries in Europe and SSA, typically uniting multiple countries in each region. Moreover, partnerships have been established extending beyond traditional colonial ties.

Concerns have been raised that African researchers are often the junior partners in research collaborations, reflected in lack of SSA lead authors on collaborative papers [17]. In this dataset, a relatively high proportion of EDCTP2 publications (72%) had lead authors from SSA, indicating that researchers from SSA play key roles in EDCTP2- funded consortia and that the program is tangibly promoting scientific leadership and excellence in the region. Moreover, although equity has not quite been achieved, women researchers from SSA were lead authors on an encouraging 33% of EDCTP2 papers indicating that EDCTP2 is enabling a relatively high proportion of women from SSA to play leading roles in research projects.

EDCTP programs take active steps to ensure active participation of women researchers in international collaborations, including in leadership positions. Representation of women authors on EDCTP2 papers is in line with the average in the field. However, representation of women authors from SSA is relatively low, reflecting the under-representation of women in research in the region [18]. A recent analysis of papers in 46 global health journals found that the proportion of women authors ranged from 45.9% in high-income countries to 28.2% in low-income countries [19], suggesting that EDCTP2 is helping to increase the representation of women from SSA on academic outputs. EDCTP2 strives for gender equity. Across its funding schemes, success rates for male and female lead applicants were comparable, but only 36.6% of successful grant applications were led by women, because women were less likely to apply as the project lead for EDCTP2 grant funding. EDCTP2 has undertaken a range of activities to address this issue, such as to train new female PhD researchers in SSA through multiple streams including through regional networks of excellence and organizing consultations to explore barriers to female participation and progression in research in Africa.

The thematic analysis has demonstrated that, compared with those from other funders, EDCTP2 outputs have a stronger focus on affordability/accessibility and on populations typically excluded from clinical research studies. This indicates that the EDCTP2 objective to focus on these issues is reflected in the research carried out.

The citation analysis indicates that EDCTP2-funded studies are generating findings of widespread importance to global health. This is particularly notable as EDCTP2 does not generally support discovery science, outputs from which are more likely to be highly cited, and has a focus on late-stage development and implementation, as well as on capacity-building, areas that tend to generate fewer academic outputs and citations [20].

The evidence of contributions of EDCTP2 outputs to policy are encouraging, although given the lag between research, publication and policy uptake, the outputs analyzed are likely to represent only a small proportion of the policy-relevant publications from the EDCTP2 program [21].

Looking ahead, multiple large EDCTP2 clinical trials are beginning to generate findings that are highly likely to inform future policy – including the CHAPAS-4 study on potential second-line antiretroviral drugs for children [22], the PediCAP trial evaluating step-down oral antibiotic treatments for severe pneumonia [23] and the CALINA trial, run by the PAMAfrica consortium, which demonstrated the safety and efficacy of a new antimalarial formulation of artemether-lumefantrine for babies weighing less than 5 kg [24].

Our findings suggest that key stakeholders – normative global agencies such as WHO, national governments and policy organizations/think tanks – are making use of EDCTP2-supported outputs and reinforce the fact that EDCTP2 has a strong practical focus on clinical challenges and policy-relevant questions, ensuring that results of funded research feed directly into (inter)national policy-making and practice.

EDCTP2 has explored ways to accelerate translation of findings into updated policy, for example by ensuring close dialogue between research teams and policymakers [21]. EDCTP2-supported researchers are encouraged to engage with the local community, policymakers and other relevant stakeholders from the start of the funding period to ensure relevance and impact of the project. In addition, EDCTP2 supported more than 50 grants focusing on implementation research relevant to the local context to narrow the gap between policy and research. Translating research findings into policy is a time-intensive process that often stretches over several years and depends on the stage of product development and contextual factors outside the direct control of researchers. Moreover, several products initially evaluated in EDCTP2 are being further evaluated in the Global Health EDCTP3.

### Limitations

This bibliometrics analysis focused on EDCTP2 publications published before the end of 2023. At this timepoint, only 53.5% of EDCTP2-supported grants had ended. Also, several EDCTP2-funded projects with high-policy impact potential were significantly delayed by the COVID-19 pandemic and their publications were not included here. The bulk of grant funding was awarded later in the program and by December 2018 only 38% of the program’s total grant funding had been committed. The final number of publications linked to EDCTP2 funding will be much higher. Similarly, there will be a further lag before findings are taken up by (inter)national policymakers.

It is challenging to determine the precise contribution of EDCTP funding to specific outputs. Many EDCTP2 publications are from large and costly late-stage trials requiring co-funding involving multiple (inter)national funders. Identification of EDCTP2-supported publications is based on mention of EDCTP2 in acknowledgements, but this does not distinguish between outputs in which the bulk of research has been funded through EDCTP2 and those in which EDCTP2-supported authors made minor contributions. Furthermore, the selection of publications included depends on the information provided by co-authors: publications lacking EDCTP2 acknowledgment were excluded.

EDCTP2 outputs were identified via the Scopus database, an abstract and citation database of peer-reviewed literature that includes more than 90 million records [25]. The database includes publications from journals that meet a set of predefined criteria relating to peer review [26]. While this process ensures the quality of publications included, it also excludes articles published in nonvetted journals. Scopus also focuses on English-language publications [27].

Overton was used as the primary source to identify policy-relevant literature citing EDCTP2 publications. Due to the timing of this analysis, the database was not yet complete for 2024. Furthermore, policy documents published by sources in sub-Saharan Africa are underrepresented [7]. Hence, this analysis considered only a subset of relevant documents within the African policy landscape.

To mitigate potential biases, wherever possible this analysis adopted a comparative approach, for example comparing EDCTP2 outputs with those acknowledging other funders, within the areas of science and medicine covered by EDCTP2.

Bibliometric indicators are a powerful tool to assess publication outputs. An earlier bibliometric analysis of EDCTP outputs showed that, for papers on poverty-related diseases, joint European–African outputs were increasing and EDCTP outputs were relatively highly cited [28]. In addition, a subsequent analysis of EDCTP outputs revealed that international collaboration and open-access publication were associated with higher citation rates [29].

A publication-based analysis provides a partial assessment of the impact of EDCTP2. Besides supporting clinical studies, EDCTP2 invested in capacity-building in sub-Saharan Africa, for example through an extensive fellowship scheme [30], capacity-building activities integrated into clinical collaborations, and specific grants to strengthen ethics review and regulatory environments. The impact of these activities is not adequately captured by bibliometric indicators and requires alternative assessment methods, including qualitative and mixed-method approaches.

Bibliometrics considers all citations equally, but high citation indices are not equivalent to high impact, as the reason for citing a paper varies. The data reported here are complementary to other sources of analysis — including yearly updates on the progress of research and capacity building activities, the content of publications, and narrative accounts of impact provided by EDCTP2 grant-holders.

## Conclusion

This bibliometric analysis, part-way through the EDCTP2 program, provides encouraging evidence that EDCTP is succeeding in its aim to strengthen and extend scientific collaborations, support equitable partnerships, ensure greater involvement of excluded populations in clinical trials, and accelerate the development and implementation of new medical interventions in SSA. As more EDCTP2-funded projects complete, this impact is expected to grow over time.

## Author contributions

DJ, AC, DB and PB conceptualized the study. HP led the analysis and created figures. DB and PB drafted the original manuscript. All authors had full access to the underlying data and reviewed and provided critical feedback on the manuscript.

## Data sharing

The findings from this study were produced using published literature only. A detailed list of EDCTP2 publications included in the analysis is available on request.

## Ethical approval

No approval was required for this analysis of previously published literature.

## Declaration of competing interest

The authors declare the following financial interests/personal relationships which may be considered as potential competing interests: Henrique Naves Pinheiro works at Elsevier since 2019, and this study is part of a commercial project that Elsevier delivered to EDCTP. No other known competing financial interests or personal relationships

## Appendix Supplementary materials

Image, application 1
